# A Miniaturized Dual-Band Frequency Selective Surface with Enhanced Capacitance Loading for WLAN Applications

**DOI:** 10.3390/s25247421

**Published:** 2025-12-05

**Authors:** Muhammad Idrees, Sai-Wai Wong, Abdul Majeed, Shu-Qing Zhang, Yejun He

**Affiliations:** State Key Laboratory of Radio Frequency Heterogeneous Integration, Sino-British Antennas and Propagation Joint Laboratory of MOST, Guangdong Engineering Research Center of Base Station Antennas and Propagation, Shenzhen Key Laboratory of Antennas and Propagation, College of Electronics and Information Engineering, Shenzhen University, Shenzhen 518060, China; muidrees169@gmail.com (M.I.); abdul@szu.edu.cn (A.M.); 2450042019@mails.szu.edu.cn (S.-Q.Z.); heyejun@126.com (Y.H.)

**Keywords:** angularly stable, electromagnetic interference (EMI), frequency selective surface (FSS), oblique incident angle, RF shielding, polarization-insensitive

## Abstract

This article presents a miniaturized dual-band frequency selective surface (FSS) based on capacitance-enhancing technique for RF shielding applications. The FSS incorporates two independent corner-modified square loop (CMSL) elements realized on a lossy dielectric, effectively suppressing the WiFi 2.45 GHz and WLAN 5.5 GHz bands simultaneously. The capacitance of FSS element is enhanced through corner truncation without using additional lumped elements. The symmetric geometry enables the FSS shield to manifest angularly stable and polarization-insensitive spectral responses under various oblique incident angles. Moreover, an equivalent circuit model (ECM) of the FSS structure is designed. A finite FSS prototype is fabricated and tested to verify the EM simulations. The measured results are in good agreement with the simulated responses. More importantly, the proposed design is scalable to other frequencies and is capable of selectively mitigating electromagnetic interference or confine the EM fields.

## 1. Introduction

Frequency selective surface (FSS) is a periodic array of resonant elements that selectively transmit or block the electromagnetic waves at specific frequencies, acting as a filter. FSSs find widespread employability in design of antenna radome [1], EM absorbers [2,3], polarization conversion [4], sub-reflector [5], antenna design [6,7], and many others. Electromagnetic shielding is an indispensable FSS application in mitigating electromagnetic interference (EMI) in sensitive environments [8,9,10,11]. Typical isolation techniques, such as metallic enclosures, wire mesh, EM absorbers, and shielding clothes, etc., can be bulky, potentially blocking all transmissions or having limited bandwidth, and are unsuitable for applications requiring multiple transmission bands. In contrast, the FSSs are regarded as a promising solution to selectively alleviating unwanted EMI.

Since achieving a dual-band or multi-band operation with a single FSS is highly advantageous, it can be accomplished either by using multiple elements or designing a single multi-resonance element. For instance, a square patch incorporating L-shaped arms [12], complementary FSS structures [13,14], a patch-slot topology [15], and closed-loop topology in [16] are investigated for dual-bandstop characteristics. These configurations reveal polarization-sensitive frequency responses with limited angular stability. Convoluted patterns are used to obtain tri-band characteristics in [17]; however, additional ripples in its spectral response degrade filtering performance for the transverse magnetic (TM) mode. In [18], an FSS shield effectively suppresses the satellite downlink frequencies. However, its SE and stop-bandwidth vary with the incident angles of the TM-polarized waves.

Moreover, a dual-element FSS design in [19] reflects the 2.4 and 5.9 GHz wireless local area network (WLAN) signals, with limited angular stability of up to only 45°. A convoluted geometry [20] obtains transmission zeros at 2.66 and 5 GHz, revealing an unstable frequency response for the transverse electric (TE) and transverse magnetic (TM) modes. Another study in [21], involving a pair of interdigitated hexapoles with intermediate tripoles, obtains two transmission zeros at 2.14 GHz and 5.13 GHz, allowing transmission at 4.4 GHz, but significant frequency shifts occur at upper stopband for TM mode with incident angle. A single-layer FSS having triple-stopband response in [22] is investigated for EMI mitigation. In [23], a dual-band shield effectively suppresses EM fields, ensuring angularly stable and polarization-insensitive responses. A polarization selective surface [24] comprising a pair of two independent tuneable square rings is studied for WLAN shielding. In addition, a modified Sierpinski fractal structure [25], superformula curves inside square loop [26], and modified square ring elements [27], are also reported for dual-band WLAN shielding applications. A convoluted connected geometry [28] filters out 5G signals at 3.4 and 4.9 GHz frequencies, offering wide angle stability up to 60°. In [11], a single-layer FSS is designed to reflect ultra-wideband (3.1–10.6) GHz frequency range. Another study [29] reports a pair of U-shaped resonators that are connected through a metallic via for dual-band, frequency, and polarization selectivity.

In the aforementioned literature, most of the reported FSSs have performance limitations in terms of polarization independence, angular stability, shielding effectiveness, and rejection bandwidth, especially for the TM polarization mode of incident EM waves. This research work is an extension of our previous article [23] and aims to present a miniaturized, dual-band-notched FSS shield that incorporates two CMSL-elements, printed on a lossy substrate. The FSS effectively mitigates interference with the following silent features. This paper makes the following key contributions:

- The anticipated FSS has a compact and simple structure, lightweight, easy fabrication, and provides a cost-effective EMI shielding solution. It outperforms the conventional square loop to accomplish miniaturization and high-angular stability.
- The FSS is designed based on a capacitance-enhancing technique without using additional lumped elements. It enables the obtaining of miniaturized cell size, high angle stability, and wider suppression bandwidth. The design achieves reflection characteristics at 2.45 GHz WiFi and 5.5 GHz WLAN bands, and a passband at about 4 GHz frequency. It achieves a size reduction of 40.2% and offers wider fractional stop-bandwidth than [23] by 13.5% and 7.0% at the lower and upper bands, respectively.
- It overcomes a well-known angular dependence issue that degrades the TM mode performance in many FSS designs [9,16,18,19,21]. The design offers independent control of both operating bands and wide oblique angle stability of up to 75°. It reveals identical spectral characteristics for the TE and TM modes, ensuring complete polarization independence, making it highly versatile in selectively mitigating the undesired EMI.

The remainder of this paper is organized as follows. Section 2 describes the design of FSS filter. Section 3 discusses the simulated results, optimization of transmission coefficient, and equivalent circuit model, including the parametric analysis. Section 4 presents the prototype fabrication, measurement setup, measured results, and shielding effectiveness. Section 5 provides a comparative analysis. Finally, Section 6 summarizes and concludes this article.

## 2. Design of Proposed FSMS

**Figure 1:**
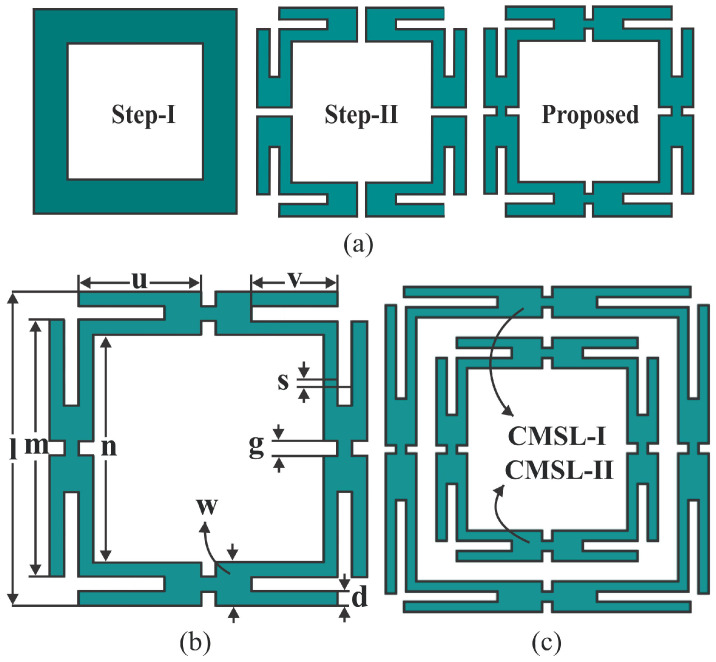
(**a**) Design procedure of the proposed FSS element. (**b**) Labeled view of CMSL-FSS element. (**c**) Top view of CMSL-I and CMSL-II elements in a co-planner arrangement.

Figure 1 illustrates the design layout of the proposed FSS band-reject filter, realized on a 1 mm thick FR-4 substrate ($\varepsilon_{r}$ = 4.4, tanδ = 0.02). The RF shield is designed based on two important factors: achieving unit cell miniaturization and obtaining an angularly stable and polarization-insensitive spectral response at both operation bands. These design constraints are addressed by considering a conventional square loop (CSL) as a stable resonant element as in Figure 1a. The CSL is divided into four segments. Each section is truncated at the corners by etching rectangular strips, which not only increases the overall electrical length but also enhances the capacitance loading and helps to achieve a miniaturized cell size. Finally, all segments are joined through the stubs to form a corner-modified square loop (CMSL) structure. The reason for considering CMSL geometry instead of the CSL is that it is a fact that loading extra inductance or capacitance brings the resonant frequency down for the same dimensions. Therefore, corner truncation adds additional capacitance to the structure and enhances the stop-bandwidth. Moreover, Figure 1b demonstrates the labeled view of the FSS single-unit element. Figure 1c specifies a co-planner arrangement of FSS elements to obtain dual-stopband spatial filter characteristics. A close integration of CMSL-I and CMSL-II results in minor variation in spectral response due to mutual coupling. The structures are further tuned to simultaneously reject the WLAN signals at 2.45 and 5.50 GHz frequencies. Thus, the unit cell is fourfold symmetric and compact with dimensions of 0.11$\lambda_{o}$ × 0.11$\lambda_{o}$ × 0.05$\lambda_{o}$. The values of optimum design variables are tabulated in Table 1.

## 3. Filtering Response of the FSMS

**Figure 2:**
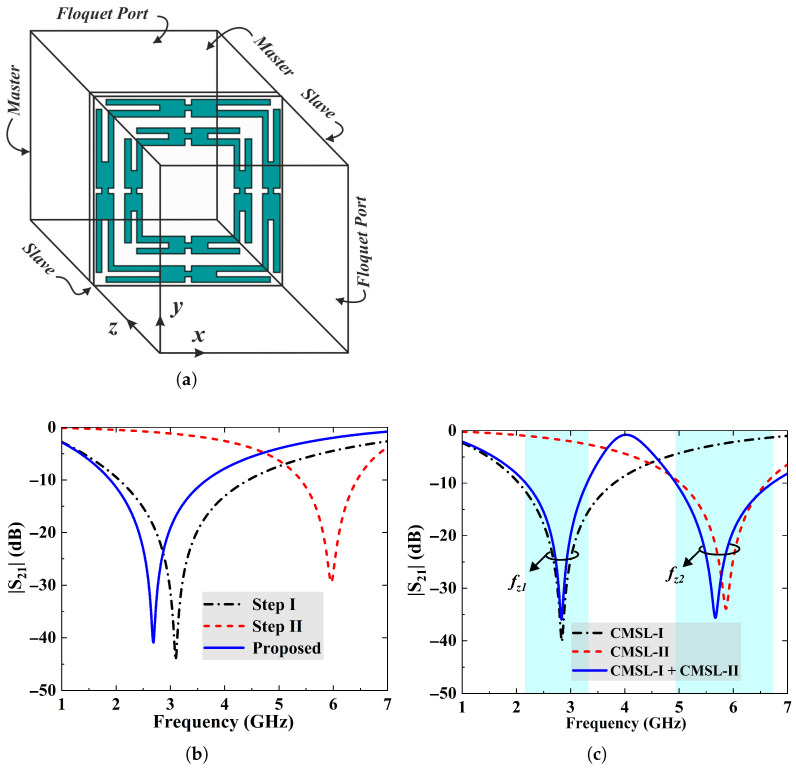
(**a**) The periodic boundary conditions setup for the FSS unit cell configured in the 3D EM simulator. (**b**) Stepwise optimization of frequency response of the FSS single-unit element. (**c**) Transmission coefficient ($\left|S_{21}\right|$) of the unit cell individual elements, and in the co-planar configuration.

This section explores the simulation and optimization of the proposed FSS geometry using Ansys HFSS, a 3D full-wave FEM-based solver. In this simulation, periodic boundary conditions (PBCs) are applied in the x and y directions, and the Floquet ports are set along the z-axis at a quarter-wavelength distance to excite the unit cell, as depicted in Figure 2a. Figure 2b shows the stepwise optimization of the transmission coefficient $\left|S_{21}\right|$ of the FSS unit element. Thus, CMSL-I resonates at the 2.85 GHz frequency. Similarly, CMSL-II is designed to operate at 5.87 GHz. Figure 2c specifies the individual and combined spectral responses of the FSS elements. The resonant frequencies of the elements vary slightly in the co-planar topology because of the mutual coupling effect. However, after further optimization, the unit cell notches at 2.45 and 5.5 GHz, while allowing transmission at about 4 GHz frequency.

### 3.1. Angle Stability and Polarization Insensitivity Analysis

**Figure 3:**
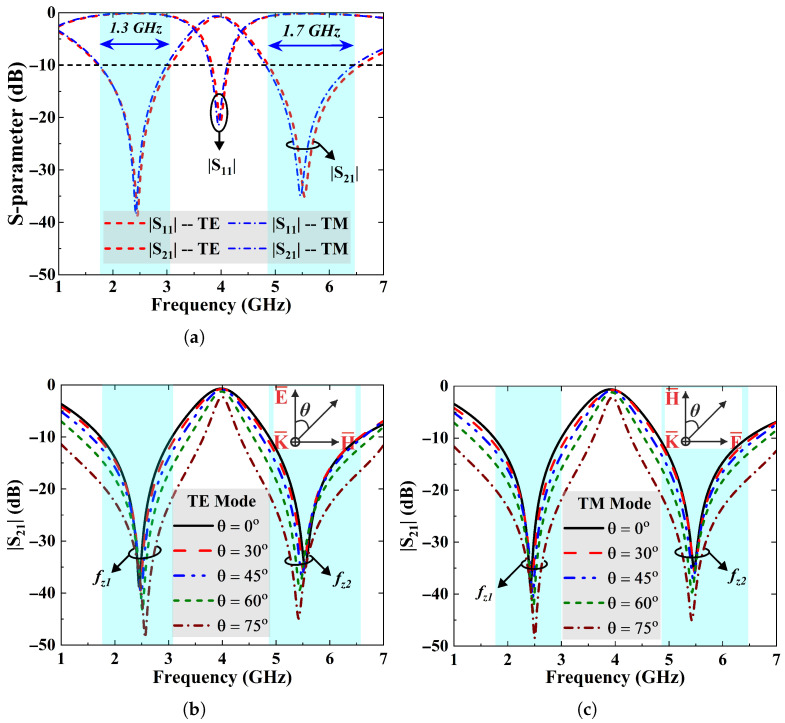
(**a**) S-parameters characteristics of the FSS unit cell at normal incidence for the TE and TM modes. (**b**) Simulated $|S_{21}|$ curves of the FSS filter confirming angular stability up to 75° for the TE-polarized wave mode. (**c**) The TM mode.

The FSS structure is subjected to various oblique incident angles and polarization states to comprehensively evaluate its electromagnetic performance. An FSS’s angular stability relies on its cell periodicity *P* within the array, as expressed in (1) [23,30].

(1) $$P<\frac{\lambda_{0}}{1+\sin(\theta )},$$

where $\lambda_{0}$ is the free-space wavelength and θ denotes the incident angle. Figure 3a shows that the simulated S-parameter responses of the FSS at normal incidence (TEz-0°) are identical for both the TE and TM polarization modes. It is observed that the FSS achieves a minimum attenuation of 38 dB and 35 dB at its notching frequencies, while offering corresponding fractional suppression bandwidths of 48.7% in the lower bands and 28.5% in the upper bands.

Moreover, Figure 3b,c present transmission coefficient $\left|S_{21}\right|$ of FSS design under varying angles of incidence for the TE and TM polarization modes. The angle of incidence is varied from 0° to 75°. It is noticed from the figures that FSS exhibits stable behavior, and angle variations do not significantly affect its resonance frequencies. Additionally, the rejection bandwidth and the notch selectivity improve as the incident angle varies. It is because of this change that FSS surface impedance with incident angle can be computed as in (2) and (3) [9,31].

(2) $$Z_{\mathrm{TE}}=\frac{Z_{{}^{\circ}}}{\cos(\theta )},$$

(3) $$Z_{\mathrm{TM}}=Z_{{}^{\circ}}\cos\left(\theta \right),$$

where $Z_{\mathrm{TE}}$ and $Z_{\mathrm{TM}}$ are the impedances experienced by the impinging TE- and TM-polarized EM waves. $Z_{{}^{\circ}}$ is the impedance of free-space whereas θ represents the incident angle between propagation vector *k* and axis of propagation. Table 2 highlights the variations in fractional stop-bandwidth and attenuation as a function of incident angle.

In addition, the stability of the stopbands can be examined using a Figure of Merit (FoM) as in (4) [9,31].

(4) $$FoM_{TE/TM}=2\times \left(\frac{FBW\left(\theta_{oblique}\right)-FBW\left(\theta_{0^{{}^{\circ}}}\right)}{FBW\left(\theta_{oblique}\right)+FBW\left(\theta_{0^{{}^{\circ}}}\right)}\right),$$

where $FBW(\theta_{0^{{}^{\circ}}})$ and $FBW(\theta_{oblique})$ represent the fractional bandwidth (FBW) at normal and oblique incidences. The FoM values close to zero indicate greater incident angle stability. The proposed FSS reveals FoM values of 0.56 and 0.49 at lower stopbands and 0.41 and 0.37 at upper stopbands for TE and TM modes, respectively.

**Figure 4:**
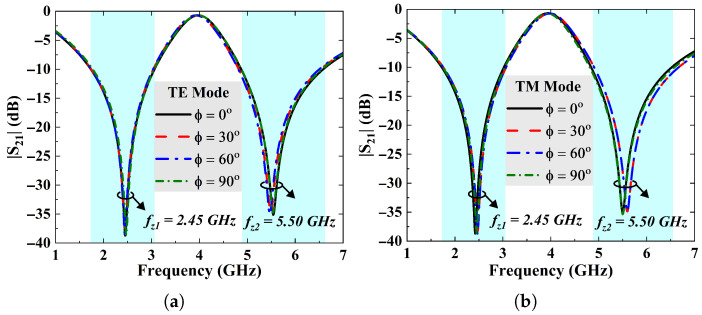
(**a**) FSS’s transmission coefficient plot as a function of varying polarization angles for TE mode and (**b**) TM mode.

Furthermore, Figure 4a,b manifest FSS’s EM behavior at normal incidence under different polarization angles. The polarization angle (ϕ) is varied from 0° to 90° at regular steps of 30°. A similar performance is observed for the TE and TM polarization states. The results indicate that overall performance of the FSS shield remains intact with variations either in the incident or polarization angles, thus confirming wide angular stability and polarization insensitivity, owing to the fourfold symmetric and compact geometry.

### 3.2. Comparison of the Proposed FSS Structure with the FSS Shield Introduced in [23]

**Figure 5:**
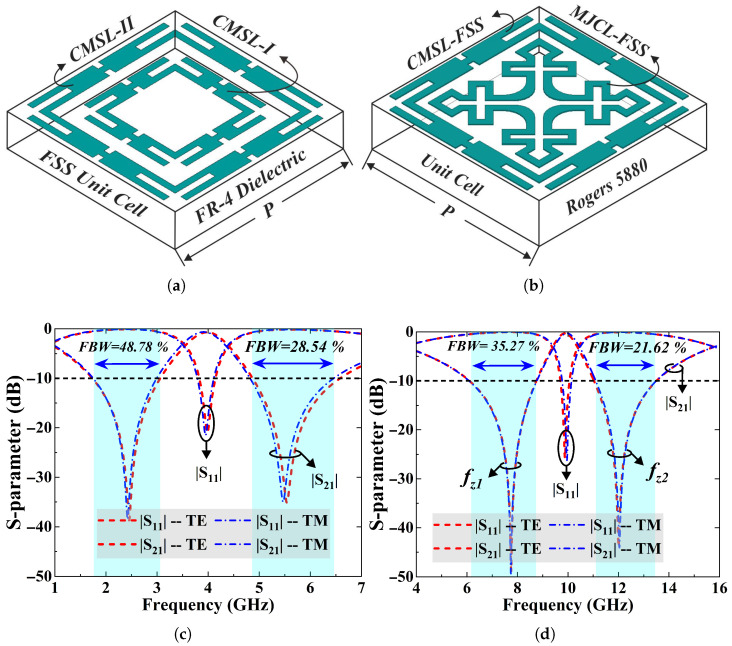
A geometric and performance-based comparison of dual-band FSS shields: (**a**) Proposed FSS structure; (**b**) FSS geometry reported in [23]. The s-parameter characteristics at normal incidence for TE and TM modes: (**c**) proposed design, (**d**) reference [23].

Figure 5 highlights a geometric and performance-based comparison of the anticipated work with an EM shield in [23]. Figure 5a shows the perspective view of the unit cell of proposed dual-band FSS filter whereas Figure 5b exhibits the structure in [23]. The proposed FSS achieves a size reduction of 40.2% compared to [23]. Moreover, Figure 5c and Figure 5d show the simulated s-parameter responses of this work and [23] at normal incidence for the TE and TM modes, respectively. The results illustrate that proposed EM shield offers wider fractional rejection bandwidth by 13.5% and 7% at its operating bands.

### 3.3. Surface Current Distribution (J-Surf) Analysis and Equivalent Circuit Model of the FSS Shield

**Figure 6:**
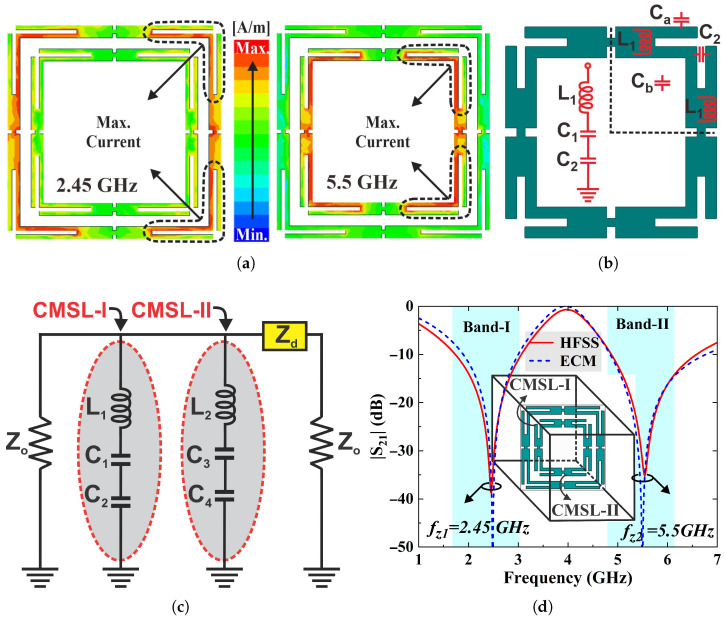
(**a**) J-surf plots of the FSS unit cell at normal incidence (TEz-0°) for the TE-polarized waves at 2.45 GHz and 5.50 GHz. (**b**) Formation of lumped elements and derivation of the circuit model of FSS unit element. (**c**) An equivalent circuit model (ECM) of dual-stopband FSS filter. (**d**) A comparison of transmission coefficient ($\left|S_{21}\right|$) of the FSS equivalent circuit and the EM models.

Thereafter, induced surface current distributions (J-surf) of the FSS are analyzed to further elucidate the connection between dual bandstop mechanism and the geometric design of the FSS. Figure 6a depicts the J-surf plots obtained at 2.5 GHz and 5.5 GHz under normal incidence for TE-polarized waves. The current distribution analysis reveals that induced currents are predominantly concentrated at the corners of FSS elements, indicating that these areas are responsible for resonance at the operating frequencies. The J-surf plots also offer insight into the formation of inductive and capacitive components to derive an equivalent circuit model (ECM). Figure 6b illustrates the formation of lumped elements on a single FSS element, where the metallic strips are modeled as inductance, while slots or gaps contribute to capacitance. As the FSS element is fourfold symmetric, one quarter of the geometry is considered for simplicity. The u-shaped metallic stub is associated with inductance $L_{1}$. The capacitance $C_{1}$ is contributed by inter-element spacing ($C_{a}$) and the capacitance inside the element ($C_{b}$) as $C_{1}$ = $C_{a}$ + $C_{b}$, while $C_{2}$ is the enhanced slot capacitance loading. The circuit model is a series combination of lumped elements. In addition, $Z_{o}=377\;\Omega$ and $Z_{d}=\frac{Z_{0}}{\sqrt{\epsilon_{r}}}$ are the characteristic impedance of free space and the substrate [23,27]. Since the FSS filter has two independent conducting geometries, a simplified ECM of FSS unit cell is a parallel combination of two LC branches as depicted in Figure 6c. The ECM is designed and simulated in the Agilent Advanced Design System (ADS). The impedance of FSS unit elements can be expressed as

(5) $$Z_{\mathrm{CMSL-I}}=j\omega L_{1}+\frac{1}{j\omega C_{1}}+\frac{1}{j\omega C_{2}},$$

(6) $$Z_{\mathrm{CMSL-I}}=\frac{\left(j\omega L_{1}\right)\left(\omega^{2}C_{1}C_{2}\right)-\left(j\omega \right)\left(C_{1}+C_{2}\right)}{\omega^{2}C_{1}C_{2}},$$

(7) $$Z_{\mathrm{CMSL-II}}=\frac{\left(j\omega L_{2}\right)\left(\omega^{2}C_{3}C_{4}\right)-\left(j\omega \right)\left(C_{3}+C_{4}\right)}{\omega^{2}C_{3}C_{4}},$$

where ω is the angular frequency. The transmission zero frequencies $f_{r1}$ and $f_{r2}$ of the FSS elements (CMSL-I and CMSL-II) can be computed as

(8) $$f_{r1}=\frac{1}{2\pi }\sqrt{\frac{C_{1}+C_{2}}{L_{1}C_{1}C_{2}}},\;\Rightarrow \;f_{r2}=\frac{1}{2\pi }\sqrt{\frac{C_{3}+C_{4}}{L_{2}C_{3}C_{4}}},$$

The overall impedance of the circuit model can be computed using (6) and (7) in (9).

(9) $$Z_{\mathrm{total}}=\frac{Z_{\mathrm{CMSL-I}}Z_{\mathrm{CMSL-II}}}{Z_{\mathrm{CMSL-I}}+Z_{\mathrm{CMSL-II}}},$$

The optimized lumped circuit parameters obtained through the curve-fitting method are tabulated in Table 3. However, different combinations of the lumped values could also achieve a similar response. Figure 6d compares the transmission coefficient curves of the ECM and EM models. The results obtained from ADS strongly align with those acquired from the HFSS. Alternatively, the transmission coefficient of the ECM can be computed using transmission line theory [32,33].

(10) $$S_{21}\left(\mathrm{dB}\right)=\frac{2Z_{0}}{AZ_{0}+B+CZ_{0}^{2}+DZ_{0}},$$

where *A*, *B*, *C*, and *D* present the coefficients of the ABCD matrix. The matrix cascading technique is applied to determine the response of cascaded structures by multiplying their individual ABCD parameters [34]. The substrate’s ABCD parameters at the normal angle of incidence can be written as

(11) $$\left[\begin{array}{cc} A_{D} & B_{D}\\ C_{D} & D_{D} \end{array}\right]=\left[\begin{array}{cc} \cos(k_{z1}t) & jZ_{0}\sin\left(k_{z1}t\right)\\ \frac{j\sin(k_{z1}t)}{Z_{D}} & \cos(k_{z1}t) \end{array}\right],$$

where *k* is the wavenumber, *t* and $z_{D}$ are the thickness and impedance of the employed dielectric substrate. The ABCD matrix of the FSS structure is expressed as

(12) $$\left[\begin{array}{cc} A_{FSS} & B_{FSS}\\ C_{FSS} & D_{FSS} \end{array}\right]=\left[\begin{array}{cc} 1 & 0\\ \frac{1}{Z_{FSS}} & 1 \end{array}\right],$$

(13) $$\left[\begin{array}{cc} A & B\\ C & D \end{array}\right]=\left[\begin{array}{cc} A_{D} & B_{D}\\ C_{D} & D_{D} \end{array}\right]\left[\begin{array}{cc} A_{FSS} & B_{FSS}\\ C_{FSS} & D_{FSS} \end{array}\right],$$

The coefficients of the ABCD matrix are used to calculate the transmission response in (10).

### 3.4. Parametric Analysis

**Figure 7:**
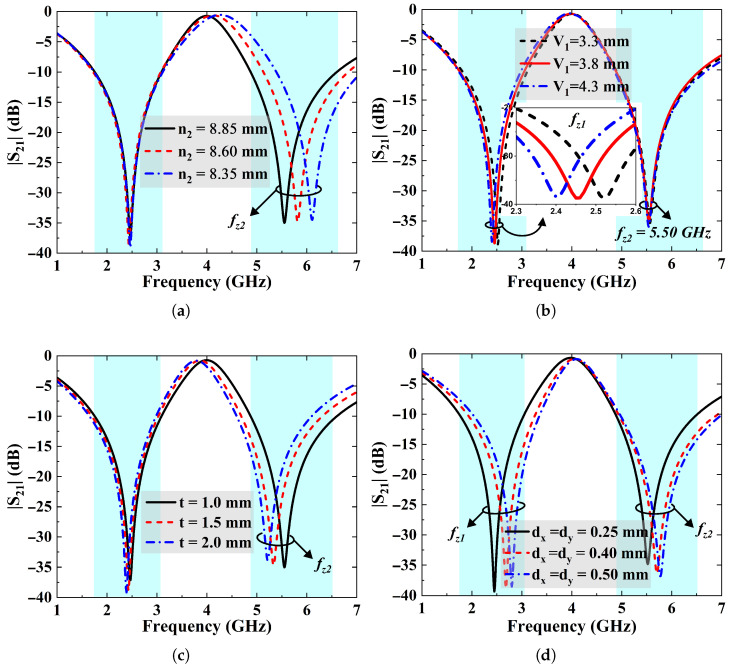
(**a**) Optimization of upper transmission zero ($f_{z2}$) as a function of perimeter of inner element CMSL-II. (**b**) Tuning of lower transmission zero ($f_{z1}$) of the FSS based on length of the slot $\left(V_{1}\right)$. (**c**) Effect of different substrate thicknesses on the resonant frequencies of the FSS. (**d**) Implications of the inter-element spacing on the position of stopbands.

In this section, a thorough parametric analysis is carried out to determine the relationship between geometric parameters and the spectral response of the anticipated FSS design. The spectral position of the transmission zeros cab be determined through parameters such as the perimeter ($n_{1}$ and $n_{2}$) and slot length ($V_{1}$ and $V_{2}$) at the corners of the FSS elements. Figure 7a shows the impact of varying the perimeter ($n_{2}$) of the FSS inner element (CMSL-II), which independently controls the upper stopband without affecting the lower band. As the value of $n_{2}$ decreases, $f_{z2}$ moves upward. Likewise, the location of the lower stopband can also be altered. However, Figure 7b specifies the impacts of varying the slot length at the corners of outer element (CMSL-I) on its frequency response. The figure illustrates that an increase in slot length ($V_{1}$) shifts the first resonance ($f_{z1}$) downward while the upper stopband remains intact. Hence, the FSS provides an independent control of its both operating bands. In addition, Figure 7c investigates the effects of laminate thickness (*t*) on the position of FSS’s operating frequencies. The figure reveals that an increase in *t* lowers the upper stopband frequency and vice versa. However, it is observed that thickness variations’ impact is more noticeable when lowering the resonance frequency of upper stopband compared to the lower stopband ($f_{z1}$), which remains almost unchanged.

Furthermore, the spacing between elements in an FSS array significantly impacts its performance. Figure 7d highlights the implications of varying the inter-element spacing ($d_{x}$ = $d_{y}$). Increasing the inter-element spacing enlarges the size of dielectric slab, which in turn raises the capacitance. This increase in capacitance lowers the resonance frequency, ultimately causing both the rejection bands to shift upwards. A large element spacing causes an early onset of grating lobes. Inter-elemental spacing also has influence on the FSS angular performance. A smaller element spacing results in higher angle stability and vice versa.

## 4. Prototype Fabrication and Experimental Verification

### 4.1. Fabrication and Test Setup

**Figure 8:**
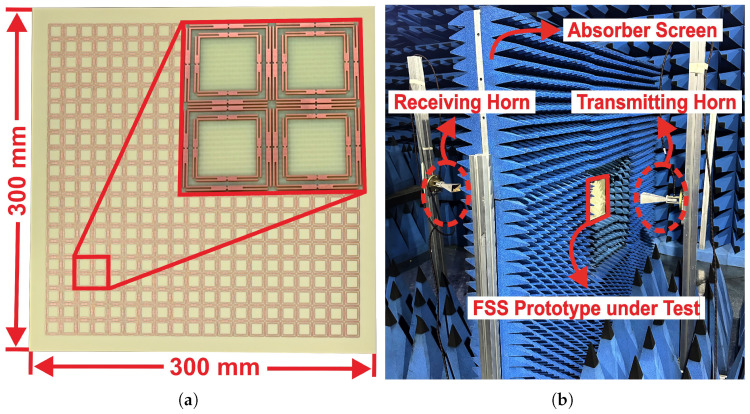
(**a**) A finite fabricated FSS panel with zoomed view of a 2 × 2 FSS array unit. (**b**) Measured S-parameter response of the fabricated FSS array for the TE and TM modes at normal incidence.

To validate the simulated design, a 20 × 20 unit cell square lattice is fabricated on an FR-4 substrate using printed circuit board (PCB) technology, as shown in Figure 8a. A photograph of a free-space measurement setup installed in an anechoic chamber [17,18,23,25], consisting of an absorber screen and a pair of horn antennas connected through the vector network analyzer (VNA), is used to measure the frequency response of the fabricated FSS. The antennas are placed at a far-field distance from the FSS panel. A prior calibration is conducted through the opening of the absorber screen without the FSS before measurements. The FSS surface is then fixed in the aperture of absorbing screen to measure its performance, as shown in Figure 8b.

### 4.2. Measurement Results

**Figure 9:**
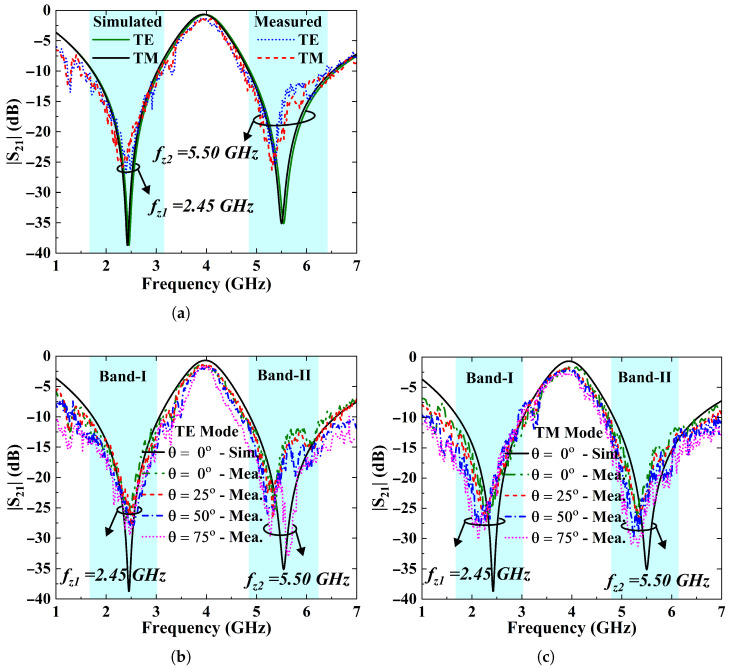
(**a**) Measured S-parameter response of the fabricated FSS array for the TE and TM modes at normal incidence. (**b**) $|S_{21}|$ curves of the FSS filter confirming angular stability up to 75° for the TE polarized wave mode. (**c**) Spectral response of the dual stopband FSS over varying incident angles ensuring stable performance for TM mode.

The transmission response of the FSS at normal incidence for the TE and TM wave modes is depicted in Figure 9a. The results reveal that the FSS has almost similar response for both polarization modes. It achieves transmission zeros at about 2.39 GHz and 5.35 GHz and offers a measured attenuation of more than 25 dB at both operating frequencies. A minor deviation of 2.45% and 2.73% in the measured transmission zero-frequencies is noted from the simulated results, which might be due to the dielectric tolerances and the cable losses.

Moreover, Figure 9b and Figure 9c demonstrate the transmission responses ($|S_{21}|$ (dB)) of the FSS surface as a function of incident angle (θ) for the TE and TM wave modes, respectively. The results indicate that FSS ensures polarization-insensitive and angularly stable responses of up to 75°. It is noticed that the fractional bandwidth and the attenuation level at both bands increase with the angle.

However, minor shifts in the transmission zero frequencies of the FSS are observed at higher incident angles, which can be calculated in (14) as

(14) $$\Delta f\left(\%\right)=\left(\frac{f_{z}-f_{\mathrm{oblique}}}{f_{z}}\right)\times 100,$$

where Δ*f*, $f_{z}$, and $f_{\mathrm{oblique}}$ are frequency shift, as well as transmission zero frequencies at normal and oblique angles, respectively. The results in Figure 9b show that the FSS surface performs steadily at the lower stopband, while the resonant frequency is shifted downwards by approximately 1.45% at the upper stopband with the angle variations for TE mode. For TM mode in Figure 9c, frequency variations of 4.81% and 2.89% have been observed for the lower and upper stopbands, respectively. The transmission loss also increases when the angle of incidence is altered from 0° to 75° for the TM mode. These minor variations in the measured results are due to the lossy dielectric material, fabrication tolerance, and measurement imperfections.

### 4.3. Shielding Effectiveness (SE)

**Figure 10:**
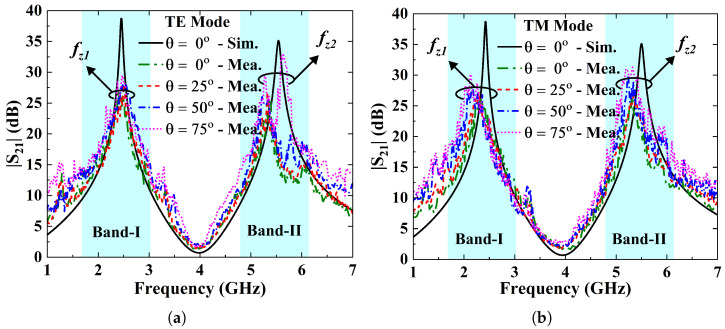
(**a**) SE of the FSS prototype against angle variations for the TE polarization mode. (**b**) SE vs. frequency curves of the FSS panel for the TM mode as a function of angle of incidence.

Shielding effectiveness (SE) quantifies how effectively an FSS shield blocks the transmission through it, often expressed in decibels (dB) and can be computed as [8,9,35]:

(15) $$\mathrm{SE}\left(\mathrm{dB}\right)=-20\log_{10}\left|\frac{E_{t}}{E_{i}}\right|=-\left|S_{21}\right|,$$

where $E_{t}$ and $E_{i}$ are the transmitted and incident electric fields, respectively. Figure 10a,b depict measured SE plots of the FSS surface under incident angle variations for the TE and TM wave modes. The design offers a measured SE of more than 25 dB at both stopbands, which further improves with incident angle for both polarizations, making it suitable for selectively mitigating the undesired EMI.

## 5. Comparison with Recent Literature

Table 4 provides a comprehensive comparison of this work with previously reported dual-band single-layer designs to assert its novelty. The comparison shows that the anticipated dual-band FSS shield is smaller in size—exhibiting angularly stable and polarization-insensitive spectral responses across varying incident angles up to 75°—than the reported studies in [13,15,21,25,27]. High angular stability is attributed to its miniaturized size and fourfold structural symmetry, which makes it suitable for selective shielding/isolation applications. In addition, the proposed design also offers independent control of each resonance while ensuring periodicity constant. It achieves wider fractional bandwidths at both the stopbands by 13.5% and 7% in comparison to the earlier work in [23], while being cost-effective as an additional feature. The FSS in [36] has larger profile in comparison to this work and reveals limited angle stability as well as a polarization-sensitive response.

Moreover, the surfaces in [21,26] also have a resonance control mechanism, but the resonances are coupled due to shared aperture geometries. Thus, altering one parameter concurrently impacts both stopbands, leading to limited angle stability and polarization-sensitive behavior. Reference [25] reports a compact, polarization-insensitive design with angle stability up to 60°, whereas the proposed FSS offers a wide angular stability up to 75°. While an FSS shield in [24] provides angularly stable and polarization-insensitive dual-band operation. However, the proposed work offers wider angle stability and rejection bandwidths at both operating frequencies.

## 6. Conclusions

This work presents a compact, polarization-insensitive FSS that achieves dual stopband characteristics utilizing enhanced capacitance loading, featuring two corner-truncated elements for the WiFi and WLAN suppression. A prototype array of 20 × 20 cells was fabricated and tested. A good correlation between the simulated and measured results is observed; the FSS manifests measured suppression of at least 25 dB at its notching frequencies. Moreover, it reveals a wide angular stability of up to 75° for TE and TM modes, ensuring complete polarization independence for the incident EM waves. Thus, the proposed design has significant application prospects to prevent signal transmission in secure environments and EMI suppression. However, in the future, we will use the same geometry to design a reconfigurable FSS.

## Figures and Tables

**Table 1:** Optimized values of the design variables of proposed FSMS unit element.

CMSL-I				CMSL-II			
Variable	Value	Variable	Value	Variable	Value	Variable	Value
d_1_	0.25	s_1_	0.25	d_2_	0.25	s_2_	0.25
g_1_	0.25	u_1_	6.0	g_2_	0.25	u_2_	6.0
l_1_	13.25	v_1_	3.8	l_2_	13.25	v_2_	3.8
m_1_	12.25	w_1_	0.75	m_2_	12.25	w_2_	0.75
n_1_	11.75	P	13.5	n_2_	11.75	d_*x*_ = d_*y*_	0.5

All the values are in mm.

**Table 2:** Attenuation level and 10 dB fractional stop-bandwidth analysis at various incident angles.

Angle (*θ*°)	Attenuation Level (dB)				Fractional Bandwidth (%)			
	Band-I		Band-II		Band-I		Band-II	
	TE	TM	TE	TM	TE	TM	TE	TM
0	38.07	38.82	35.26	35.41	48.78	51.35	28.52	28.87
30	38.85	39.91	35.83	35.76	54.17	56.36	30.04	30.56
45	40.24	41.27	36.98	37.24	62.57	67.78	32.94	34.72
60	42.93	43.76	39.47	39.74	80.33	84.97	39.14	40.84
75	48.35	49.13	44.87	44.68	120.53	123.07	49.95	51.34

**Table 3:** Optimized values of lumped parameters.

CMSL-I			CMSL-II		
$L_{1}$	$C_{1}$	$C_{2}$	$L_{2}$	$C_{3}$	$C_{4}$
1.12 nH	6.9 pF	7.8 pF	0.72 nH	2.32 pF	2.34 pF

**Table 4:** Comparison of proposed dual-band-reject FSS with closely related existing FSS designs in the literature for EM shielding applications.

Ref. No.	Unit Cell Periodicity	Substrate Material	Operating Bands	SE (dB) Band-I/II	FBW (%) Band-I/II	Angle Stability	TM Mode Stability	Polarization Insensitive
[13]	0.16$\lambda_{o}$ × 0.16$\lambda_{o}$	FR-4	2.5/5.5	35/30	35/30	45°	Yes	Yes
[15]	0.33$\lambda_{o}$ × 0.33$\lambda_{o}$	FR-4	2.41/5.71	NR	NR	30°	N/A	NR
[19]	0.11$\lambda_{o}$ × 0.11$\lambda_{o}$	FR-4	2.4/5.9	38/32	18/10.3	45°	N/A	Yes
[21]	0.25$\lambda_{o}$ × 0.25$\lambda_{o}$	PET and Paper	2.1/5.1	22/20	24.5/9.4	45°	N/A	NR
[23]	0.18$\lambda_{o}$ × 0.18$\lambda_{o}$	Rogers 5880	7.8/11.9	48.7/49.0	35.2/21.6	75°	Yes	Yes
[24]	0.06$\lambda_{o}$ × 0.06$\lambda_{o}$	FR-4	2.5/5.45	32/33	24/21.6	60°	Yes	Yes
[25]	0.12$\lambda_{o}$ × 0.12$\lambda_{o}$	Float glass	2.4/5.4	N/A	39.6/30.5	60°	N/A	NR
[27]	0.20$\lambda_{o}$ × 0.20$\lambda_{o}$	Polyester	2.4/5.0	35/42	20.7/13.6	60°	N/A	Yes
[36]	0.31$\lambda_{o}$ × 0.31$\lambda_{o}$	FR-4	2.4/5.2	45/43	NR	45°	N/A	NR
This Work	0.11$\lambda_{o}$ × 0.11$\lambda_{o}$	FR-4	2.45/5.5	38.1/35.2	48.7/28.5	75°	Yes	Yes

Where $\lambda_{o}$ is the free-space wavelength at the first resonant frequency. N/A: Not Appreciable; NR: Not reported.

## Data Availability

Data are contained within the article.
